# Sex-stratified mortality trends in preterm birth complications in Sierra Leone: progress, persistence, and equity implications

**DOI:** 10.1080/16549716.2026.2713866

**Published:** 2026-08-06

**Authors:** Alpha Umaru Bai-Sesay, Rosetta Doreen Jones, Alimamy Paul Simeon Baun, Daniel Karim Dauda Sesay

**Affiliations:** a Ministry of Health/National Public Health Agency, Freetown, Sierra Leone; b Research and Scientific Division, Sustainable Health Systems, Freetown, Sierra Leone; c Department of Accident and Emergency, 34 Military Hospital, Wilberforce, Freetown, Sierra Leone; d Research and Scientific Division, Insights for Development Impact, Freetown, Sierra Leone

**Keywords:** Neonatal mortality, preterm birth complications, health equity, sex‑based disparities, Sierra Leone, WHO HEAT metrics, inequality monitoring, neonatal policy

## Abstract

**Background:**

Preterm birth complications remain a leading cause of neonatal mortality in Sierra Leone, despite recent health system gains. Evidence on long-term sex-specific disparities in mortality due to preterm birth complications is limited, constraining equitable neonatal care planning.

**Objective:**

To examine two‑decade trends in sex‑stratified mortality from preterm birth complications using standardized equity indicators.

**Methods:**

We conducted a retrospective longitudinal analysis of sex-disaggregated mortality estimates from the World Health Organization (WHO) Global Health Estimates (GHE), accessed through the WHO Health Equity Assessment Toolkit (HEAT), Built-in Database Edition (Version 6.0). Mortality rates per 100,000 population were extracted for 2001, 2006, 2011, 2016, and 2021. Inequality was assessed using absolute difference (*D*), relative ratio (*R*), population attributable risk (PAR), and population attributable fraction (PAF).

**Results:**

Mortality declined substantially between 2001 and 2021 for both males (85.1–49.3 per 100,000) and females (71.2–39.9 per 100,000). Male mortality remained consistently higher across all years, with relative ratios indicating approximately 20–25% excess mortality among male neonates. Absolute inequalities narrowed modestly over time, whereas relative inequalities remained largely unchanged. PAR and PAF remained close to zero throughout the study period. Wider uncertainty intervals in earlier years reflected limited empirical data availability.

**Conclusion:**

Although preterm mortality declined over two decades, a persistent male disadvantage remained in Sierra Leone. These findings highlight the importance of integrating sex-disaggregated equity monitoring into neonatal policies and programmes. Future research should evaluate strategies to reduce the persistent excess mortality among male neonates while sustaining overall improvements in neonatal survival and progress toward Sustainable Development Goal 3.2.

## Paper Context

- **Main findings:** Mortality from preterm birth complications fell substantially in Sierra Leone between 2001 and 2021, but male neonates consistently experienced about 20–25% higher mortality than females.
- **Added knowledge:** This is the first two-decade, sex-stratified analysis of preterm mortality in Sierra Leone using standardized WHO equity metrics, revealing a persistent male disadvantage that remained stable despite overall survival gains.
- **Global health impact for policy and action:** Routine sex-disaggregated monitoring and sex-sensitive neonatal care protocols are essential to ensure that progress toward SDG 3.2 is equitable and does not leave male preterm infants behind.

## Background

Preterm birth complications, defined as births occurring before 37 completed weeks of gestation, remain the leading cause of neonatal mortality worldwide, accounting for more than 1 million deaths annually and disproportionately affecting low- and middle-income countries (LMICs) [1,2]. Sierra Leone continues to experience one of the highest neonatal mortality rates globally, reflecting longstanding challenges including limited neonatal intensive care capacity, fragile health information systems, and post-conflict health system rebuilding [3,4]. Understanding how this burden is distributed among population subgroups is essential for achieving equitable reductions in neonatal mortality under Sustainable Development Goal (SDG) 3.2 and the Every Newborn Action Plan [5].

While global evidence consistently documents higher mortality among male neonates, sex-disaggregated analyses of preterm mortality remain limited in many LMIC settings, including Sierra Leone. The biological vulnerability of male preterm infants, characterized by delayed lung maturation, increased susceptibility to respiratory distress, and higher risk of infectious and neurological complications is well established [6,7]. However, these biological risks do not operate in isolation [8]. They interact with health-system constraints, such as inconsistent access to oxygen therapy and temperature regulation, sociocultural dynamics that shape care-seeking behavior, and inequities in facility readiness for managing preterm complications [9–11]. Thus, sex functions not only as a biological variable but also as a WHO-recognized equity stratifier, making sex-stratified mortality analysis essential for identifying avoidable and unfair disparities [12].

Sierra Leone presents a relevant case study due to its persistent burden of preterm mortality, ongoing maternal and newborn health reforms, and efforts to strengthen its health information infrastructure following the Ebola epidemic. However, trend analyses are often limited by sparse data inputs and inconsistent disaggregation. The World Health Organization’s Health Equity Assessment Toolkit (HEAT) provides standardized, publicly accessible, and comparable estimates that allow countries with historically weak civil registration and vital statistics systems to monitor inequities over time. Although modeled estimates have important limitations, HEAT enables systematic sex-disaggregated assessment using harmonized methods when high-quality primary data are unavailable.

HEAT includes a suite of equity indicators, such as absolute difference, relative ratio, population attributable risk (PAR), and population attributable fraction (PAF) that together describe both the magnitude and proportional direction of disparities. These indicators have been widely applied in global inequality monitoring. However, longitudinal analyses applying these standardized equity metrics to track sex-based disparities in preterm mortality are scarce for high-burden countries like Sierra Leone. This leaves a critical gap in context-specific evidence needed to determine whether Sierra Leone’s disparity patterns are consistent with regional trends and to inform equity-oriented policy.

In this study, we examine sex-stratified mortality trends due to preterm birth complications in Sierra Leone from 2001 to 2021 using WHO Global Health Estimates accessed through HEAT. Our objectives are to (1) describe long-term changes in male and female neonatal mortality, (2) quantify sex-based disparities using standardized equity indicators, and (3) interpret findings within the biological, structural, and health-system factors that shape neonatal outcomes. This analysis aims to strengthen equity-oriented monitoring of neonatal health and inform sex-sensitive clinical and policy responses necessary to accelerate progress toward SDG 3.2 in high-burden settings.

## Methods

### Study design and data sources

We conducted a retrospective, longitudinal analysis of sex-stratified neonatal mortality due to preterm birth complications in Sierra Leone from 2001 to 2021. The study followed the Strengthening the Reporting of Observational Studies in Epidemiology (STROBE) guidelines and WHO Protocols for monitoring health inequalities. Mortality estimates were obtained from the WHO Global Health Estimates (GHE), accessed through the WHO Health Equity Assessment Toolkit (HEAT), Built-in Database Edition (Version 6.0). GHE combines inputs from civil registration and vital statistics (CRVS) systems, demographic and health surveys, censuses, and disease-specific surveillance using standardized modeling procedures. Although modeled data have important limitations in settings with historically weak CRVS systems, such as Sierra Leone, they currently represent the only nationally comparable, sex-disaggregated, cause-specific neonatal mortality estimates available for long-term analysis.

### Study population and stratification

The study population comprised neonates aged 0–27 days, stratified by sex (male and female). Mortality rates were expressed per 100,000 total population, consistent with WHO GHE reporting conventions. Sex disaggregation followed WHO’s recommended equity stratifiers.

### Selection of time points

Five reference years were selected: 2001, 2006, 2011, 2016, and 2021. These points align with HEAT’s structured, 5-year interval data architecture and allow examination of long-term shifts while minimizing the noise, instability, and year-to-year fluctuations inherent in modeled estimates, particularly in earlier years where uncertainty intervals are wider. These time points also correspond to major policy periods, including the Millennium Development Goals (2000–2015) and Sustainable Development Goals (2016–2030). The use of 5-year intervals is consistent with the presentation of long-term trends in WHO HEAT and reduces the influence of short-term fluctuations in modelled annual estimates, thereby facilitating more robust interpretation of changes in mortality and inequality over time.

### Measures and analytical approach

Mortality due to preterm birth complications (World Health Organization Global Health Estimates cause-specific category) was extracted for each sex and selected time point using the WHO Health Equity Assessment Toolkit (HEAT), Built-in Database Edition (Version 6.0). HEAT was used to calculate four WHO-standardized inequality indicators: Absolute difference (*D*): female mortality rate minus male mortality rate, as reported by the WHO Health Equity Assessment Toolkit (HEAT). Negative values indicate higher mortality among male neonates. Relative ratio (*R*): male mortality rate divided by female mortality rate. Population Attributable Risk (PAR): excess mortality that would be prevented if the disadvantaged subgroup had the same mortality as the reference. Population attributable fraction (PAF): the proportion of total mortality attributable to the observed disparity.

In accordance with WHO HEAT guidance, both absolute (difference [*D*] and population attributable risk [PAR]) and relative (ratio [*R*] and population attributable fraction [PAF]) summary measures were reported because they provide complementary information on the magnitude and relative distribution of health inequalities. Where available, 95% confidence intervals generated by HEAT were reported for all summary measures. For binary sex comparisons, HEAT does not generate confidence intervals for *D* and *R*; therefore, only point estimates are reported for these metrics.

All inequality calculations were performed directly by the HEAT software using its built-in algorithms; no additional statistical adjustments or post-processing were applied to the extracted mortality rate estimates.

PAR and PAF are presented because they quantify the population‑level burden attributable to the disparity, which is useful for policy prioritization. However, in binary stratification with nearly equal population sizes and modest absolute differences, these values are expected to be close to zero. They should therefore be interpreted alongside absolute and relative measures, which are more informative for sex‑based comparisons.

### Ethical considerations

This study was carried out in accordance with the principles of the Declaration of Helsinki. The analysis used publicly available, anonymized, aggregated data and required no ethical approval. All procedures adhered to WHO data‑use policies and national guidelines for secondary analysis.

## Results

**Figure 1.**
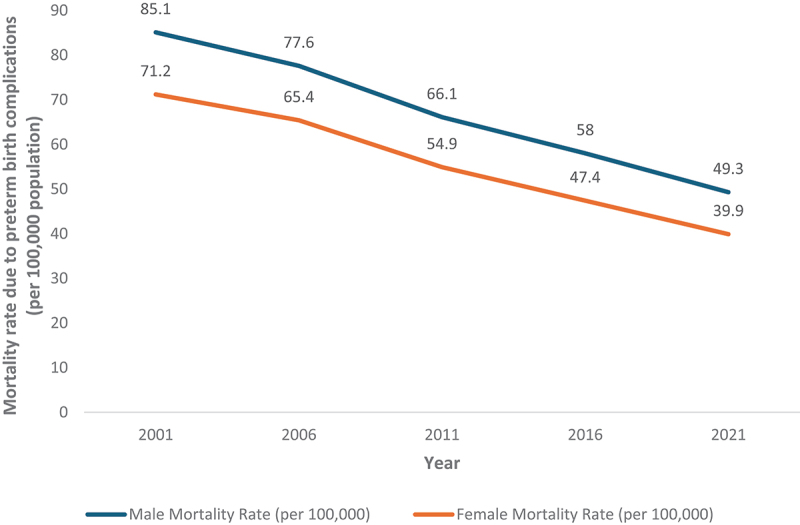
Sex-stratified mortality rates due to preterm birth complications in Sierra Leone, 2001–2021. Mortality rates are expressed per 100,000 population using WHO Global Health estimates accessed through HEAT Version 6.0. Shaded areas represent 95% confidence intervals.

Between 2001 and 2021, mortality due to preterm birth complications declined steadily among both male and female neonates in Sierra Leone, although mortality remained consistently higher among males throughout the study period (Table 1; Figure 1). Among male neonates, mortality decreased from 85.1 per 100,000 population (95% CI: 56.6–124.5) in 2001 to 49.3 (95% CI: 29.6–73.9) in 2021. Female mortality declined from 71.2 per 100,000 (95% CI: 47.4–104.2) to 39.9 (95% CI: 23.9–59.8) over the same period.

**Table 1.** Mortality rates due to preterm birth complications in Sierra Leone by sex, 2001–2021.

Year	Sex	Mortality rate (per 100,000)	95% CI (Lower–Upper)	Population estimate
2001	Female	71.2	47.4–104.2	2,444,289
	Male	85.1	56.6–124.5	2,412,807
2006	Female	65.4	38.3–88.5	2,914,009
	Male	77.6	45.4–104.9	2,895,766
2011	Female	54.9	35.1–78.1	3,307,516
	Male	66.1	42.4–93.9	3,304,869
2016	Female	47.4	30.8–65.6	3,742,430
	Male	58	37.8–80.3	3,751,484
2021	Female	39.9	23.9–59.8	4,201,801
	Male	49.3	29.6–73.9	4,218,841

Note: ^a^Mortality rates are expressed per 100,000 total population, as reported by WHO Global Health Estimates (GHE) via HEAT Version 6.0.

^b^Population estimates are rounded to the nearest whole number.

^c^CI = 95% confidence interval.

Data source: WHO Global Health Estimate s accessed through the Health Equity Assessment Toolkit (HEAT), Built-in Database Edition Version 6.0.

Male mortality exceeded female mortality at all time points. The absolute difference (male minus female mortality) narrowed modestly from 13.9 per 100,000 in 2001 to 9.4 in 2021, reflecting gradual convergence in absolute terms.

The relative ratio (male:female mortality) remained approximately 1.2–1.25, indicating that male neonates consistently experienced about 20–25% higher mortality than female neonates over the two-decade period (Table 2).

**Table 2.** Sex-based inequality metrics for preterm birth mortality in Sierra Leone, 2001–2021.

Year	D (95% CI)	R (95% CI)	PAR (95% CI)	PAF (95% CI)
2001	−13.9 (NA)	1.2 (NA)	0 (−2.5 to 2.5)	0 (0–0)
2006	−12.1 (NA)	1.2 (NA)	0 (−2.2 to 2.2)	0 (0–0)
2011	−11.2 (NA)	1.2 (NA)	0 (−1.9 to 1.9)	0 (0–0)
2016	−10.6 (NA)	1.2 (NA)	0 (−1.6 to 1.6)	0 (0–0)
2021	−9.4 (NA)	1.2 (NA)	0 (−1.4 to 1.4)	0 (0–0)

Note: ^a^Absolute difference (*D*) is defined as female mortality minus male mortality, as calculated by HEAT. Negative values indicate higher mortality in males.

^b^Confidence intervals for *D* and *R* are not generated by HEAT for binary sex comparisons; therefore, only point estimates are reported.

^c^PAR and PAF are shown with 95% CIs from HEAT.

Data source: WHO Global Health Estimates, accessed through the Health Equity Assessment Toolkit (HEAT), Built-in Database Edition Version 6.0.

Population attributable risk (PAR) and population attributable fraction (PAF) remained close to zero in all years. This pattern is expected in two-group comparisons with relatively small absolute differences and equal population distribution between groups. Low PAR and PAF values do not imply that sex disparities are unimportant; rather, they reflect the mathematical properties of attribution measures under binary stratification and should be interpreted alongside absolute and relative measures.

Uncertainty intervals were wider in earlier years (e.g. in 2001, male 95% CI: 56.6–124.5; female: 47.4–104.2), reflecting sparse input data and greater model uncertainty. By 2021, confidence intervals narrowed, indicating improved data completeness and stability of underlying population estimates. These trends mirror broader improvements in Sierra Leone’s health information systems following national investments in post-Ebola recovery and routine surveillance strengthening.

## Discussion

This study examined two decades of sex-stratified mortality due to preterm birth complications in Sierra Leone, revealing substantial overall declines in mortality but persistent and patterned male disadvantage. These findings align with global evidence showing that male neonates experience higher mortality risks, yet they also highlight how biological susceptibility interacts with health-system weaknesses, sociocultural dynamics, and structural inequities in low-resource settings. The stability of the relative disparity (*R* ≈ 1.2) over two decades, despite overall mortality declines, mirrors patterns observed in other sub-Saharan African settings with similarly constrained neonatal care systems [13–17], suggesting this may be a characteristic of settings where biological vulnerability is amplified by systemic limitations. Together, these intersecting factors contribute to the consistent 20–25% higher male mortality observed throughout the study period [4,18,19].

The overall reductions in preterm-related mortality reflect incremental improvements in Sierra Leone’s maternal and newborn health system following major investments during the Millennium Development Goals era, the introduction of neonatal-focused policies through the Every Newborn Action Plan, and post-Ebola health system strengthening [4]. The observed decline in mortality coincided with expansions in skilled birth attendance, infection prevention capacity, Kangaroo Mother Care (KMC), and the availability of basic neonatal care. Although causal relationships cannot be established in this descriptive analysis, these health-system improvements may have contributed to the observed reductions in mortality [20,21]. However, these improvements were insufficient to reduce sex-based inequities, which remained stable in proportional terms even as absolute mortality declined [22].

Male neonates are biologically more vulnerable to complications such as respiratory distress syndrome, intraventricular hemorrhage, and sepsis due to delayed lung maturation, higher rates of surfactant deficiency, and immunological immaturity [23]. While these mechanisms underpin excess male mortality globally, their effects are amplified in settings where neonatal care resources remain extremely limited. Constraints such as inconsistent access to oxygen therapy, suboptimal thermal regulation, limited use of bubble CPAP, inadequate monitoring capacity, and shortages of specialized neonatal staff disproportionately affect infants with the highest physiological vulnerability, predominantly male preterm newborns.

However, biological vulnerability alone does not fully explain the persistent relative disparity. System-level factors, such as late referral, delayed recognition of respiratory distress, workload constraints limiting close monitoring, and gaps in consistent application of KMC may differentially affect preterm male infants [4,9,10]. Sociocultural dynamics may also shape care-seeking patterns and immediate postnatal responses, although systematic evidence for sex-differentiated care-seeking in Sierra Leone remains limited [21]. These biological, health-system, and sociocultural factors may collectively contribute to the persistence of the observed relative disparity in male-to-female mortality despite overall improvements in neonatal survival.

Throughout the study period, PAR and PAF values remained close to zero. This pattern must be interpreted correctly: low PAR/PAF values in a binary comparison reflect the mathematical properties of attribution measures when absolute differences are modest and the population is nearly equally distributed across categories, as is the case for sex. These low values do not imply that sex-based disparities are trivial or clinically unimportant. Instead, absolute differences and relative ratios provide more meaningful insight into sex-based inequities in this context. Accurate interpretation of these indicators is critical to equity-focused neonatal policy.

### Policy and programmatic implications

The persistent male disadvantage in preterm mortality, evidenced by a stable 20–25% higher relative risk (*R* ≈ 1.2) over two decades, underscores that general improvements in neonatal care have been insufficient to close a biologically anchored equity gap. This stable disparity, measured through standardized WHO equity metrics, necessitates that neonatal policies and clinical protocols move beyond uniform approaches to explicitly integrate sex-based vulnerability. We propose the following priorities, sequenced from direct, data-driven actions to forward-looking considerations.

First, the consistent disparity quantified in this analysis directly mandates the integration of routine sex-disaggregated monitoring into national neonatal surveillance systems. The necessity for tools like the Health Equity Assessment Toolkit transitions from research to routine program management; district health teams should use these metrics to track disparity trends and evaluate the equity impact of interventions. This foundational step ensures that progress toward Sustainable Development Goal 3.2 is measured not just by aggregate survival but by equitable survival across subgroups.

Second, the quantified excess mortality risk supports the adoption of sex-sensitive clinical risk stratification within national newborn care guidelines. Specifically, the well-documented biological vulnerabilities of male preterm infants to respiratory and infectious complications, which underlie the observed disparity can be addressed through targeted, resource-conscious protocols. These include enhanced respiratory monitoring and a lower threshold for initiating bubble CPAP in the first 48–72 h for male preterm infants, prioritized thermal regulation support, and routine early sepsis screening. Such measures operationalize the evidence of heightened risk without requiring substantial new resources.

Third, strengthening foundational newborn care with an explicit equity lens is critical. Quality improvement efforts for Kangaroo Mother Care (KMC), basic respiratory support, and reliable oxygen therapy must be designed and audited to ensure they reach the most physiologically vulnerable infants, who are disproportionately male. Audit mechanisms should track the coverage and quality of these interventions by infant sex, ensuring these life-saving practices are mitigating, not inadvertently perpetuating, the observed disparity.

Looking forward, addressing the broader structural and behavioral barriers that shape neonatal outcomes is essential. Investments in emergency referral systems, transportation, and community-level recognition of preterm danger signs will benefit all newborns but may disproportionately benefit male infants, whose biological susceptibility can lead to more rapid deterioration following any delay in care-seeking.

Finally, the potential of emerging, context-appropriate innovations should be explored. While our data cannot evaluate specific technologies, the persistent gap highlights the potential value of innovations like low-cost, IoT-enabled monitoring devices for early detection of respiratory compromise. Strategic piloting of such technologies in high-burden facilities could provide evidence for their role in mitigating specific vulnerabilities, but they must complement not replace investments in skilled human resources and essential medical commodities.

### Strengths and limitations

This study has several strengths. It provides a two-decade, sex-disaggregated analysis of mortality due to preterm birth complications in Sierra Leone, an area with limited prior documentation, despite a high neonatal mortality burden. The use of standardized WHO Health Equity Assessment Toolkit indicators offers a transparent and internationally comparable approach for monitoring disparities, enabling interpretation within globally recognized equity frameworks. Consistent use of harmonized definitions and time points enhances comparability across years, while adherence to STROBE guidelines strengthens methodological rigor.

However, important limitations must be acknowledged. First, the analysis is based on modeled estimates from the WHO Global Health Estimates, which rely on statistical synthesis of disparate data sources. In Sierra Leone, where civil registration and vital statistics systems, facility reporting, and cause-of-death certification have historically been incomplete, these modeled estimates carry substantial uncertainty, particularly in earlier years. Wider uncertainty intervals in early periods reflect sparse empirical inputs and should be interpreted cautiously.

Second, the modeled GHE estimates, particularly for earlier years, are derived from sparse and potentially unrepresentative input data. Underreporting of neonatal deaths, especially early deaths at home or in poorly equipped facilities is widespread in Sierra Leone. While such underreporting likely affects mortality estimates for both sexes, any systematic difference in care-seeking, referral, or registration practices by infant sex could bias the estimated magnitude of the disparity. Furthermore, the lack of data on key clinical and social confounders within HEAT, such as gestational age subcategories, birth weight, or socioeconomic status, prevents an intersectional analysis that could elucidate the compounded drivers of these observed disparities.

Third, as a retrospective observational analysis, the study cannot establish causal relationships. Unmeasured factors, including variations in care-seeking behavior, referral patterns, facility readiness, and community practices may influence observed disparities but could not be assessed with the available data. Missing data from home deliveries, early neonatal deaths occurring outside health facilities, and inconsistent documentation may further contribute to misclassification or underestimation of mortality.

Despite these limitations, the study offers a valuable equity-oriented assessment of sex-based disparities in preterm mortality in a high-burden, data-constrained setting. The findings should be interpreted as indicative of broad patterns rather than precise point estimates, reinforcing the need for improved neonatal surveillance systems and investment in high-quality, sex-disaggregated primary data.

### Future directions

Future research should prioritize facility-based or population-level data to validate modeled estimates and reduce uncertainty. Intersectional analyses incorporating socioeconomic status, maternal education, and geographic access could reveal compounded inequities, informing targeted interventions. Evaluating the effectiveness of sex-sensitive neonatal care protocols, such as enhanced respiratory support for male infants, would provide evidence for scalability in low-resource settings. Integrating HEAT into routine national surveillance could sustain equity monitoring, aligning with global health commitments. Exploring structural and behavioral factors, such as health system capacity, care-seeking delays, and gender norms, would further elucidate drivers of disparities, guiding comprehensive policy responses.

## Conclusion

Over the past two decades, Sierra Leone has made meaningful progress in reducing mortality due to preterm birth complications, yet persistent and measurable sex-based disparities remain. Male neonates consistently experienced approximately 20–25% higher mortality than female neonates throughout the study period. Although the Population Attributable Risk (PAR) and Population Attributable Fraction (PAF) remained close to zero, the persistent relative inequality and residual absolute differences indicate that improvements in neonatal survival have not eliminated sex-based disparities in mortality due to preterm birth complications.

These findings highlight the importance of incorporating sex-disaggregated equity monitoring into neonatal health policies and programmes to ensure that reductions in mortality are achieved equitably. Routine integration of sex-disaggregated data into national health information systems, together with the continued use of equity monitoring tools, such as the WHO Health Equity Assessment Toolkit (HEAT), can support evidence-informed decision-making, accountability, and ongoing assessment of progress toward reducing neonatal health inequalities. Future implementation research should evaluate strategies that effectively reduce the persistent excess mortality among male preterm neonates in resource-constrained settings.

Strengthening neonatal care with an explicit equity lens is essential for accelerating progress toward Sustainable Development Goal 3.2 and ensuring that reductions in neonatal mortality benefit all newborns in Sierra Leone.

## Supplementary Material

strobe checklist proper.pdf

## Data Availability

The data analyzed in this study are publicly available through the World Health Organization Health Equity Assessment Toolkit (HEAT), Built-in Database Edition (Version 6.0), which provides access to the World Health Organization Global Health Estimates (GHE): https://whoequity-heat-1.share.connect.posit.cloud/

## References

[1] Getaneh T, Homaira N, Kasaye H, et al. Global inequities in the survival of extremely preterm infants: a systematic review and meta-analysis. BMC Pediatr. 2025;25:579. doi: 10.1186/s12887-025-05933-w 40739629 PMC12309206

[2] Bradley E, Blencowe H, Moller A-B, et al. Born too soon: global epidemiology of preterm birth and drivers for change. Reprod Health. 2025;22:105. doi: 10.1186/s12978-025-02033-x 40551192 PMC12186353

[3] Sierra Leone Child Survival Action Plan. pdf. 2023–2025 [cited 2025 Aug 3]. Available from: https://www.childhealthtaskforce.org/sites/default/files/2024-03/Sierra%20Leone%20Child%20Survival%20Action%20Plan%202023-2025.pdf

[4] Osborne A, Bai-Sesay AU, Bangura C, et al. Socio-economic and geographical inequalities in neonatal mortality rates in Sierra Leone, 2008–2019. BMC Pediatr. 2024;24:761. doi: 10.1186/s12887-024-05189-w 39578763 PMC11585221

[5] WHO. Every newborn: an action plan to end preventable deaths. [cited 2026 Jul 18]. Available from: https://www.who.int/publications/i/item/9789241507448

[6] Mahtab S, Madhi SA, Baillie VL, et al. Causes of death identified in neonates enrolled through Child Health and mortality prevention surveillance (CHAMPS), December 2016–December 2021. PLOS Glob Public Health. 2023;3:e0001612. 36963040 10.1371/journal.pgph.0001612 PMC10027211

[7] Nabila M, Baidani A, Mourajid Y, et al. Analysis of risk determinants of neonatal mortality in the Last Decade: a systematic literature review (2013–2023). Pediatr Rep. 2024;16:696–8. doi: 10.3390/pediatric16030059 39189293 PMC11348196

[8] Gilson L, Lehmann U, Schneider H. Practicing governance towards equity in health systems: LMIC perspectives and experience. Int J Equity Health. 2017;16:171. doi: 10.1186/s12939-017-0665-0 28911320 PMC5599870

[9] Moxon SG, Lawn JE, Dickson KE, et al. Inpatient care of small and sick newborns: a multi-country analysis of health system bottlenecks and potential solutions. BMC Pregnancy Childbirth. 2015;15:S7. doi: 10.1186/1471-2393-15-S2-S7 26391335 PMC4577807

[10] Lawn JE, Mwansa-Kambafwile J, Horta BL, et al. ‘Kangaroo mother care’ to prevent neonatal deaths due to preterm birth complications. Int J Epidemiol. 2010;39:i144–i154. doi: 10.1093/ije/dyq031 20348117 PMC2845870

[11] Nkuranga JB, Hadfield BR, Bukeyeneza J, et al. An evaluation of oxygen therapy and respiratory support practices among hospitals in the African neonatal network. J Afr Neonatol. 2025;3:114–119. doi: 10.4314/jan.v3i3.8

[12] Hosseinpoor AR, Nambiar D, Schlotheuber A, et al. Health equity Assessment Toolkit (HEAT): software for exploring and comparing health inequalities in countries. BMC Med Res Methodol. 2016;16:141. doi: 10.1186/s12874-016-0229-9 27760520 PMC5069829

[13] Moges N, Dessie AM, Anley DT, et al. Burden of early neonatal mortality in sub-Saharan Africa. A systematic review and meta-analysis. PLOS ONE. 2024;19:e0306297. doi: 10.1371/journal.pone.0306297 39052580 PMC11271883

[14] Mabrouk A, Abubakar A, Too EK, et al. A scoping review of preterm births in sub-Saharan Africa: burden, risk factors and outcomes. Int J Environ Res Public Health. 2022;19:10537. doi: 10.3390/ijerph191710537 36078258 PMC9518061

[15] Yaya S, Diarra S, Mabeu MC, et al. The sex gap in neonatal mortality and the AIDS epidemic in sub-saharan Africa. BMJ Glob Health. 2018;3:e000940. doi: 10.1136/bmjgh-2018-000940 PMC6135478 30233834

[16] Lingappan K, Alur P, Eichenwald E. The need to address sex as a biological variable in neonatal clinical studies. J Pediatr. 2023;255:17–21. doi: 10.1016/j.jpeds.2022.11.021 36460079 PMC10416542

[17] Alur P, Holla I, Hussain N. Impact of sex, race, and social determinants of health on neonatal outcomes. Front Pediatr. 2024;12:1377195. doi: 10.3389/fped.2024.1377195 38655274 PMC11035752

[18] Johnson JO. Implementation of family centered care for neonates admitted in a special care baby unit in Sierra Leone. Pediatr Health Med Ther. 2024;15:189–199. doi: 10.2147/PHMT.S455804 PMC11107938 38774023

[19] Townsel CD, Emmer SF, Campbell WA, et al. Gender differences in respiratory morbidity and mortality of preterm neonates. Front Pediatr. 2017;5:6. Epub ahead of print 30 January 2017. DOI: 10.3389/fped.2017.00006 28194395 PMC5276811

[20] Bell NVT, Luke RDC, Akhigbe IE, et al. Knowledge, attitude, and practice of kangaroo mother care among neonatal nursing staff at ola during children’s hospital, freetown, sierra leone. Sierra Leone J Biomed Res. 2024;15. Available from: https://www.sljbr.org/index.php/sjbmr/article/view/186, (accessed 3 August 2025).

[21] Ameyaw EK, Dickson KS. Skilled birth attendance in Sierra Leone, Niger, and Mali: analysis of demographic and health surveys. BMC Public Health. 2020;20:164. doi: 10.1186/s12889-020-8258-z 32013896 PMC6998232

[22] Sserwanja Q, Mufumba I, Kamara K, et al. Rural–urban correlates of skilled birth attendance utilisation in Sierra Leone: evidence from the 2019 Sierra Leone demographic Health survey. BMJ Open. 2022;12:e056825. doi: 10.1136/bmjopen-2021-056825 PMC8961150 35351721

[23] Melville JM, Moss TJM. The immune consequences of preterm birth. Front Neurosci. 2013;7:79. doi: 10.3389/fnins.2013.00079 23734091 PMC3659282

