# Supplementary material for: Sex-stratified mortality trends in preterm birth complications in Sierra Leone: progress, persistence, and equity implications
Source: Glob Health Action. 2026 Aug 6;19(1):2713866. doi: 10.1080/16549716.2026.2713866 (PMC13449426; doi:10.1080/16549716.2026.2713866)
Supplement: Supplementary file 1 [file ZGHA_A_2713866_SM6171.pdf]

STROBE Statement—checklist of items that should be included in reports of observational studies

Item No.	Recommendation	Response (with page references)
**Title and Abstract**		
**1(a)**	Indicate the study's design with a commonly used term in the title or abstract.	**Page 1:** Title describes mortality trends. **Page 2 (Abstract):** States that the study was a **retrospective longitudinal analysis** using WHO HEAT data.
**1(b)**	Provide an informative and balanced abstract.	**Page 2:** Abstract includes background, objective, methods, results and conclusions.
**Introduction**		
**2**	Explain the scientific background and rationale.	**Pages 3–4:** Describes the burden of preterm birth complications, evidence of sex differences in neonatal mortality, and the importance of monitoring inequalities using WHO HEAT.
**3**	State specific objectives and any prespecified hypotheses.	**Page 4:** Objectives are clearly stated: (1) examine temporal trends, (2) quantify sex inequalities using WHO HEAT measures, and (3) interpret findings within biological and health-system contexts. No prespecified hypothesis was tested.
**Methods**		
**4**	Present key elements of study design early in the paper.	**Page 4:** Study described as a retrospective longitudinal observational study based on secondary WHO HEAT/GHE data.
**5**	Describe the setting, locations and relevant dates.	**Page 4:** National-level study conducted in Sierra Leone using WHO Global Health Estimates for 2001, 2006, 2011, 2016 and 2021. No recruitment or follow-up was undertaken because secondary aggregated data were analysed.
**6(a)**	Describe eligibility criteria and participant selection.	**Page 5:** The study analysed national aggregate mortality estimates for neonates (0–27 days) stratified by sex. Individual participant selection was not performed because publicly available secondary data were used.
**6(b)**	Matching criteria (if applicable).	**Not applicable.** No matching was performed.
**7**	Clearly define outcomes, exposures, predictors, confounders and effect modifiers.	**Page 5:** Outcome: mortality due to preterm birth complications (per 100,000 population). Exposure/stratifier: sex (male/female). Diagnostic definitions followed WHO Global Health Estimates. No confounders or effect modifiers were available.
**8**	Data sources and measurement.	**Pages 4–5:** Data were obtained from WHO HEAT Version 6.0 using the WHO Global Health Estimates database. Mortality estimates were generated using standardized WHO modelling methods, ensuring comparability across sex groups.
**9**	Describe efforts to address bias.	**Pages 5–6 and 10–11:** Potential bias from modelled estimates, incomplete civil registration and uncertainty was acknowledged. Standardized WHO methods reduced measurement inconsistency.
**10**	Explain study size.	**Page 5:** All available national WHO HEAT estimates for the five reporting years were included. No sample-size calculation was required because the complete available dataset was analysed.
**11**	Explain handling of quantitative variables.	**Page 5:** Mortality rates were analysed as continuous variables expressed per 100,000 population. Sex remained binary as provided by the WHO dataset.
**12(a)**	Describe statistical methods.	**Pages 5–6:** WHO HEAT was used to estimate Difference (D), Ratio (R), Population Attributable Risk (PAR) and Population Attributable Fraction (PAF) with 95% confidence intervals. No confounder adjustment was possible because aggregated data lacked covariates.
**12(b)**	Describe subgroup analyses.	**Pages 5–6:** Analyses were stratified by sex only. No interaction analyses were conducted.

Item No.	Recommendation	Response (with page references)
**12(c)**	Explain how missing data were addressed.	**Page 6:** Individual-level missing data were not applicable. WHO Global Health Estimates use standardized modelling approaches to address incomplete underlying data.
**12(d)**	Explain analytical methods accounting for sampling strategy (if applicable).	**Not applicable.** Sampling was not performed because national modelled estimates were analysed.
**12(e)**	Describe sensitivity analyses.	**Not applicable.** No sensitivity analyses were undertaken.
**Results**		
**13(a)**	Report numbers of individuals at each stage.	**Page 7 (Table 1):** National male and female population estimates and mortality rates for each reporting year are presented. No participant recruitment or exclusions occurred.
**13(b)**	Give reasons for non-participation.	**Not applicable.**
**13(c)**	Flow diagram.	**Not applicable.**
**14(a)**	Describe participant characteristics.	**Page 5:** Sex was the only available stratifying variable. Individual demographic and clinical characteristics were unavailable in the aggregate dataset.
**14(b)**	Report missing data.	**Page 6:** No variable-specific missing data existed within the published WHO HEAT estimates. Limitations related to underlying source data are acknowledged.
**14(c)**	Summarise follow-up time.	**Not applicable.**
**15**	Report outcome events or summary measures.	**Pages 7–8:** Table 1 and Figure 1 present mortality rates (per 100,000 population) with 95% confidence intervals across all years for males and females.
**16(a)**	Report estimates and precision.	**Pages 7–8:** Unadjusted mortality rates and inequality measures with 95% confidence intervals are reported. No adjusted analyses were possible because confounding variables were unavailable.
**16(b)**	Report category boundaries.	**Not applicable.** Continuous variables were not categorised.
**16(c)**	Translate relative risk into absolute risk (if relevant).	**Not applicable.**
**17**	Report other analyses.	**Pages 7–8:** Sex-based inequality analyses using Difference, Ratio, PAR and PAF were performed. No additional subgroup or sensitivity analyses were conducted.
**Discussion**		
**18**	Summarise key results.	**Pages 8–9:** Discusses declining mortality over time while persistent male disadvantage remained.
**19**	Discuss limitations.	**Pages 10–11:** Limitations include reliance on modelled estimates, uncertainty due to incomplete vital registration, lack of individual-level variables, inability to adjust for confounding and inability to infer causality.
**20**	Provide overall interpretation.	**Pages 8–11:** Findings are interpreted in relation to biological vulnerability, health-system improvements, previous literature and study limitations.
**21**	Discuss generalisability.	**Page 11:** Findings are likely applicable to similar low-resource settings with weak civil registration systems but may not generalise to countries with complete vital registration.
**Other Information**		
**22**	Give funding source and role of funders.	**Page 14:** States that the study received **no specific funding** from any public, commercial or not-for-profit funding agency.

Continued on next page
